# Prediction of Specific TCR-Peptide Binding From Large Dictionaries of TCR-Peptide Pairs

**DOI:** 10.3389/fimmu.2020.01803

**Published:** 2020-08-25

**Authors:** Ido Springer, Hanan Besser, Nili Tickotsky-Moskovitz, Shirit Dvorkin, Yoram Louzoun

**Affiliations:** ^1^Department of Mathematics, Bar Ilan University, Ramat Gan, Israel; ^2^The Goodman Faculty of Life Sciences, Bar Ilan University, Ramat Gan, Israel

**Keywords:** TCR repertoire analysis, epitope specificity, evaluation methods, machine learning, deep learning, long short-term memory (LSTM), autoencoder (AE)

## Abstract

Current sequencing methods allow for detailed samples of T cell receptors (TCR) repertoires. To determine from a repertoire whether its host had been exposed to a target, computational tools that predict TCR-epitope binding are required. Currents tools are based on conserved motifs and are applied to peptides with many known binding TCRs. We employ new Natural Language Processing (NLP) based methods to predict whether any TCR and peptide bind. We combined large-scale TCR-peptide dictionaries with deep learning methods to produce ERGO (pEptide tcR matchinG predictiOn), a highly specific and generic TCR-peptide binding predictor. A set of standard tests are defined for the performance of peptide-TCR binding, including the detection of TCRs binding to a given peptide/antigen, choosing among a set of candidate peptides for a given TCR and determining whether any pair of TCR-peptide bind. ERGO reaches similar results to state of the art methods in these tests even when not trained specifically for each test. The software implementation and data sets are available at https://github.com/louzounlab/ERGO. ERGO is also available through a webserver at: http://tcr.cs.biu.ac.il/.

## Introduction

T lymphocytes (T cells) are pivotal in the cellular immune response (1, 2). The immense diversity of the T-cell receptor (TCR) enables specific antigen recognition (3, 4). Successful recognition of antigenic peptides bound to Major Histocompatibility Complexes (pMHCs) requires specific binding of the TCR to these complexes (5–7), which in turn modulates the cell's fitness, clonal expansion, and acquisition of effector properties (7). The affinity of a TCR for a given peptide epitope and the specificity of the binding are governed by the heterodimeric αβ T-cell receptors (2). While both chains have been reported to be important to affect binding, we show here that for many TCR-peptide pairs the TCR's binding to target MHC-peptide can be determined with high accuracy using the β-chain only. Including the alpha chain in the analysis is essential for better accuracy. However, as many experimental settings provide, only beta chains, a binding prediction tool based on these chains is of importance.

Within the TCRβ chain, the complementarity-determining region 1 (CDR1) and CDR2 loops of the TCR contact the MHC alpha-helices while the hypervariable complementary determining regions (CDR3) interact mainly with the peptide (1, 2). In both TCRα and TCRβ chains, CDR3 loops have the highest sequence diversity and are the principal determinants of receptor binding specificity.

Following specific binding of T cell receptors to viral and bacterial-derived peptides bound to MHC (5), or from neo-antigens (8–10), the appropriate T cells expand, resulting in the increased frequency of T cells carrying such receptors. Recently, high-throughput DNA sequencing has enabled large-scale characterization of TCR sequences, producing detailed T cell repertoire (Rep-Seq) (11). Expanded clones are more likely to be repeatedly sampled in Rep-Seq than non-expanded clones and can serve as biomarkers for previous or current exposures to their cognate target, so tools that precisely identify TCRs binding different targets are essential in utilizing T cell repertoires as systemic biomarkers (often referred to as “reading the repertoire”).

A direct method for using TCR Rep-Seq as biomarkers has been proposed by Emerson et al. (12) and similar approaches (13) who detected patients that have Cytomegalovirus (CMV) based on their full repertoire and the choice of TCRs that differ between CMV positive and negative patients (12, 14). This approach is based on the presence of highly specific and repetitively observed public TCR in the response of different hosts to the same peptide [often denoted public clones, although the definition of such clones varies among authors (15)]. Such an approach requires extensive repertoire sequencing for every condition tested.

In contrast, many TCR responses are characterized by a high level of cross-reactivity with single TCRs binding a large number of MHC-bound peptides, and single peptides binding a large number of TCRs (16, 17). TCRs binding the same MHC-peptide may share similarities. Thus, while for public clones the task of deciphering the relation between a peptide and the TCR binding is based on tallying the candidate public TCR, for most highly cross-reactive TCRs, a probabilistic approach is required.

Important steps have been made in this direction by Glanville et al. (4) and Dash et al. (18), who detected the clear signature of short amino acid motifs in the CDR3 region of TCRβ and TCRα in response to specific peptides presented by specific MHC molecules. This work was then extended by recent efforts that combined these motifs with machine learning to predict peptide-specific TCRs vs. naïve TCRs, using Gaussian Processes (19) or Random Forest (20), or predicting TCR-epitope binding with Convolutional Neural Networks (21, 22). These methods significantly outperform random classification in the distinction of TCR binding a specific peptide and random TCRs.

All the approaches above are sequence-based and do not model the structure of the interaction. Moreover, it is assumed that the TCR-peptide binding is a binary prediction, instead of explicitly computing the off-rate or on-rate. This is indeed a simplification, based on an arbitrary cutoff of the affinity. Also, all such predictions ignore cross-reactivity; no attempt is made to predict whether a given peptide is the only target of a TCR (or vice versa).

Still, this question is of importance in two experimental setups. The first case is the attempt to predict from deep sequencing whether a given host has been infected by a pathogen [e.g., CMV (12, 14)]. A similar question that is often raised is which receptors to test for a given target. The binary solution to this question helps to sort TCRs to test. For both scenarios, predicting whether a TCR binds a peptide is of importance. We here follow a similar approach and propose a clear framework for the validation of current approaches, and a novel method to predict the binding of any peptide to any TCR (instead of predicting binding to predefined peptide).

The next required step for using the repertoire to develop specific biomarkers would be to distinguish between TCR binding different peptides. An essential step in the development of high precision predictors is the standardization of the comparison methods.

In contrast with most machine learning tasks, where one attempts to predict the output for a given input (e.g., predicting the content of an image), TCR binding is a pairing problem, where one is given a pair of inputs X and Y (a peptide and a TCR), and the goal is to predict whether they would bind. As such, there are many ways to divide the train and the test, and as a result many possible tests. One could for example assume that either X or Y are fixed and already seen in the training phase, and the other is varied. An alternative division could be that all X and Y are already known during the training and the test is on whether a given pair of known X and Y would bind. Finally, one could imagine a more complex scenario where one would ask on X and Y both absent from the training set whether they bind.

**Figure 1:**
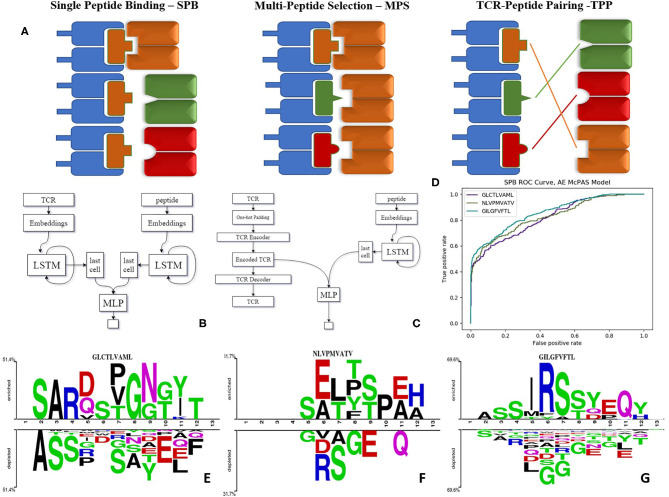
**(A)** Illustration of the tests we suggest for evaluating the model performance as explained—SPB, single peptide binding; MPS, multi-peptide selection; and TPP, TCR-peptide pairing. **(B)** LSTM based model architecture. **(C)** Autoencoder based model architecture. **(D)** ROC curve of autoencoder based model SPB performance on 3 human peptides from Dash et al. (18) dataset. **(E–G)** Comparison of amino acids of CDR3 beta sequences of TCRs binding Dash et al. (18) peptides vs. TCRs that do not bind these peptides, in McPAS (23) database (logos were created with Two-Sample-Logos); the height of symbols within the stack in each logo indicates the relative frequency of each amino acid at that position. Only amino acids whose distribution differs significantly between the two sets are shown, and only 13 length TCRs were compared.

This formally translates into five different tests, each with different outcomes, as the standard method to estimate such predictions (Figure 1A):

- Single Peptide Binding—SPB. Testing whether an unknown TCR binds a predefined target, using (as training information) TCRs known to bind to this target (18–20). In other words, the target is fixed, and TCRs are divided into disjoint training and test sets. The outcome of such a prediction would be the Area Under Curve (AUC) for the binding of an unseen TCR to this target.
- Multi-Peptide Selection—MPS. Given a set of predefined peptides, predict which of those will be bound by a new TCR. In such a case, one trains on a set of different target peptides, and TCRs are again divided into disjoint training and test sets. The outcome of that would be the accuracy of the choice as a function of the number of candidate peptides.
- TCR-Peptide Pairing I—TPP-I. Given a large set of peptides and TCRs, test whether a randomly chosen TCR binds a randomly chosen peptide. In this task, all TCR and peptides both belong to training and test sets. However, TCR-peptide pairs are divided into disjoint training and test sets.
- TCR-Peptide Pairing II—TPP-II is similar to TPP-I, except that now, TCRs contained in the pairs belonging to the training set cannot belong to the test set.
- TCR-Peptide Pairing III—TPP-III is again a similar test on pairs, but here neither TCR nor peptide can be in both training and test set.

We propose these different tests as standard measures for the quality of TCR-peptide binding predictions. The TCR-peptide pairing (TPP) task is often addressed in Natural Language Processing (NLP) using recurrent neural networks (RNN) (24). Long short-term memory (LSTM) networks are common types of RNN (25). We employed LSTMs that produce an encoding of the varying TCR and peptide into constant length real-valued encodings and created ERGO (pEptide tcR matchinG predictiOn). In all the following results, ERGO is only trained for the TPP-I task and tested on all other tasks.

## Results

### ERGO Outline

Target peptides and TCRs have different generation mechanisms [TCRs through VDJ recombination and junctional diversity (11), and peptides through antigen generation, trafficking, processing and MHC binding (26)]. As such they have different sequence probability distributions. To capture these differences, ERGO uses different parallel encoders. At the broad level, we encode the CDR3 of each TCR and each peptide into numerical vectors. The encoded CDR3 and peptide are concatenated and used as an input to a feed-forward neural network (FFN), which is trained to output 1 if the TCR and peptide bind and 0 otherwise (Figures 1B,C). At this stage, the MHC and V genes were not included since they did not contribute significantly to prediction accuracy in the current formalism. We plan to further enlarge the formalism to include both.

For the peptides, we first use an initial random embedding and translated each amino acid (AA) into a 10-dimensional embedding vector. Changing the encoding dimension did not significantly change the obtained accuracy. To merge the encoding vectors of each position into a single vector representing the peptide, each vector was used as input to an LSTM. We used the last output of the LSTM as the encoding of the whole sequence. The embedding matrix values, the weights of the LSTM, and the weights of the FFN were trained simultaneously. For the TCR encoding, we either used a similar approach or an autoencoder (AE) (see Methods and Figure 1C).

These models were trained on two large datasets of published TCR binding specific peptides (23, 27). McPAS-TCR (23) is a manually curated database of TCR sequences associated with various pathologies and antigens based on published literature, with more than 20,000 TCRβ sequences matching over 300 unique epitope peptides. These TCRs are associated with various pathologic conditions (including pathogen infections, cancer, and autoimmunity) and their respective antigens in humans and mice. VDJdb (27) is an open, comprehensive database of over 40,000 TCR sequences and over 200 cognate epitopes and the restricting MHC allotype acquired by manual processing of published studies. For each TCR-peptide pair, a record confidence score was computed. ERGO was trained on both CD4 and CD8 T cell receptors. However, the large majority of peptides and TCR in both McPAS and VDJdb are CD8 T/MHC-I TCR/peptide combinations (Supplementary Figure S1C). When testing ERGO on TPP-I-III, we used an equal distribution of CD4 and CD8 T cell receptors in the training and test sets.

### ERGO Can Predict TCR Binding to Specific Epitopes or Antigens (SPB Task)

ERGO was trained to solve the TPP-I problem (pairing TCR and peptide) on the two datasets above, and then tested on all five mentioned tests. To test the performance of ERGO on the SPB task (detecting whether a previously unseen TCR binds a known peptide), we analyzed the five most frequent peptides in each dataset and tested the possibility of detecting whether a randomly selected TCR binds the peptide. The AUC for the binary classifications ranged between 0.695 and 0.980 (Table 1). The results are not sensitive to the number of TCRs reported for the peptides, and all peptides with more than 50 reported TCRs had similar values (Supplementary Table S4).

**Table 1:** Comparison between the different versions of the ERGO classifier [AE (Autoencoder) vs. LSTM and McPAS (23) vs. VDJdb (27)] for the SPB task.

**Peptide**	**McPAS**		**Peptide**	**VDJdb**	
	**AE**	**LSTM**		**AE**	**LSTM**
LPRRSGAAGA	0.772	0.760	KLGGALQAK	0.695	0.731
GILGFVFTL	0.843	0.832	GILGFVFTL	0.820	0.817
NLVPMVATV	0.835	0.821	NLVPMVATV	0.665	0.686
GLCTLVAML	0.803	0.816	AVFDRKSDAK	0.676	0.695
SSYRRPVGI	0.969	0.980	RAKFKQLL	0.828	0.825

*The five most frequent peptides in each database are shown. Other less frequent peptides SPB results are in the Supplementary Table S4. The values are the AUC over the test set of a previously unseen TCR for this peptide*.

Note that ERGO is never trained on any specific target. Instead, it learns a model for the entire set of peptides through the LSTM. As such, its performance on different peptides varies and is a function of the fit of the trained model to this specific peptide. This is both a strength and a weakness of ERGO. It is a strength in that it applies to a wide range of peptides, but a weakness since for a specific peptide with a large number of known binding TCRs, it can perform worse than existing classifiers.

To compare ERGO to current approaches, we tested its performance on current tools that predict TCR-peptide binding. We first compared it to the work of Jokinen et al. (19) who compared TCRs found by Dash et al. (18) to bind three human epitopes and seven mice epitopes with TCRs from VDJdb database (27), which bind additional 22 epitopes. These peptide-TCR pairs were compared with naïve TCRs not expected to recognize the epitopes. Jokinen et al. evaluated the TCRGP model using leave-one-subject-out cross-validation (LOSO). The TCRGP model was trained with all subjects but one at a time and tested on the last. In the VDJdb data, the authors use 5-fold cross-validation instead of LOSO. Other evaluations were reported by using leave-one-out cross-validation of all unique TCRs (as defined by CDR3 sequence and V-gene). We compared ERGO when only the CDR3β sequence is utilized with the published TCRGP results for three specific human peptides from Dash et al. (18) dataset. ERGO outperforms TCRGP models on 2/3 peptides, although ERGO was not trained to solve the SPB task for these specific peptides, but rather the more generic TPP task. ERGO was also compared to another epitope-specific based model by Gielis et al. (20), that used random forest algorithm to train their TCRex model. Gielis et al. distinguished between epitope-specific TCRs from McPAS (23) and VDJdb (27) databases (after some filtering methods) and background TCRs that were taken from an external dataset. Unlike ERGO, the TCRex data usage also includes the V and J genes. Nevertheless, ERGO performance on the SPB task is competitive with TCRex results on various peptides, even though ERGO is trained without V and J genes on the TPP task (Table 2 and Figure 1D, Supplementary Table S5 and methods for details of the training and test procedure for these and all other tests).

**Table 2:** Comparison between the different versions of the ERGO classifier [AE vs. LSTM and McPAS (23) vs. VDJdb (27)] and existing classifiers [TCRGP by Jokinen et al. (19), TCRex by Gielis et al. (20)] for the SPB task.

**Peptide**	**ERGO**						**ERGO best**	**TCRGP (β,3), LOSO**	**TCRGP (β,3), unique LOO**	**TCRex**
	**McPAS**		**VDJdb**		**McPAS+ VDJdb**					
	**AE**	**LSTM**	**AE**	**LSTM**	**AE**	**LSTM**				
GLCTLVAML	0.803	0.816	0.764	0.770	0.708	0.686	0.816	0.782	**0.852**	0.82 ± 0.02
NLVPMVATV	0.835	0.821	0.665	0.686	0.624	0.632	**0.835**	0.587	0.651	0.72 ± 0.01
GILGFVFTL	0.843	0.832	0.820	0.817	0.725	0.712	**0.843**	0.818	0.822	0.81 ± 0.01

*Bolded values are the best results. The peptides here are the human peptides in Dash et al. (18) dataset proposed by Jokinen et al. (19). The other VDJdb peptides tested by the same authors are in Supplementary Table S5. The values are the AUC over the test set of previously unseen TCR for this peptide*.

We used two-sample-logos (28) to compare the CDR3 sequences of cognate TCRs for the three human peptides from Dash et al. (18) dataset with TCRs that do not bind these peptides in the McPAS database (23) (Figures 1E–G). Only 13 AA long TCRs were compared to avoid any alignment bias. While one can see that different peptides have different signatures, it is interesting to see that the signature is not equally positioned among peptides. For the GLCTLVAML peptide, a signature is divided equally along the TCR, with a strong bias for the initial “CSA” at the beginning of the CDR3 sequence and not “CAS.” The NLVPMVATV signature is distributed following the standard “CAS” to the end of the CDR3, while the GILGFVFTL binding peptides are characterized by a dominant RS at position 6-7. Note that a part of this difference can be the result of different V and J gene usage, which is not explicit in ERGO, but may be captured by the algorithm.

The single peptide binding task can be extended to the single antigen protein task, where we predict whether a TCR would bind any peptide from a protein. Instead of testing whether an unseen TCR can bind a specific peptide, we tested whether it can bind any peptide from a target protein. The performance on this task varies drastically between target peptides, with AUC ranging from 0.71 to 0.97 (Table 3). This difference is not directly related to the number of target TCRs in the training set, but may rather represent the contribution of other factors not incorporated here, such as the alpha chain or the MHC.

**Table 3:** Comparison between the different versions of the ERGO classifier [AE vs. LSTM and McPAS (23) vs. VDJdb (27)] for the binding to a specific antigen.

**Protein**	**McPAS**		**Protein**	**VDJdb**	
	**AE**	**LSTM**		**AE**	**LSTM**
NP177	0.772	0.767	IE1	0.703	0.738
M1	0.843	0.832	M	0.825	0.820
pp65	0.814	0.803	pp65	0.702	0.716
BMLF1	0.808	0.819	EBNA4	0.711	0.717
PB1	0.958	0.970	Gag	0.890	0.897

*There are no previous results on this task*.

### Determining the Target of a TCR (MPS Task)

**Figure 2:**
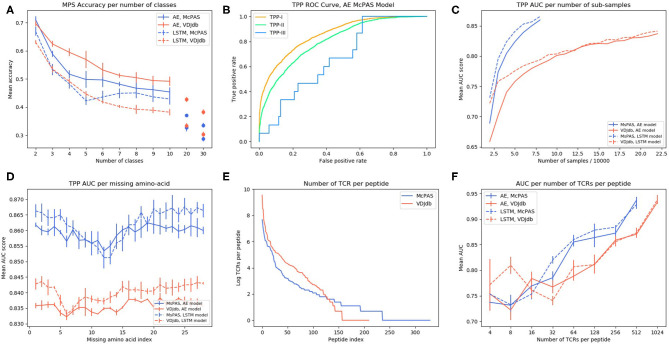
**(A)** AE and LSTM models MPS accuracy per number of peptide classes in McPAS-TCR (23) and VDJdb (27) datasets. **(B)** ROC curve of TPP-I, II, and III AE model performance on McPAS dataset. **(C)** AUC for TPP-I as a function of the sub-sample size. **(D)** AUC of TPP-I per missing amino-acids index. **(E)** Number of TCRs per peptide distribution in McPAS-TCR and VDJdb datasets, logarithmic scale. **(F)** AUC of TPP-I per number of TCRs per peptide bins (bins are the union of all TCRs that match peptides with total number of TCRs in a specific range).

To use a TCR as a biomarker, one should be able to predict which specific peptide it binds. To test for that, we computed the accuracy (as measured by the sum of the diagonal in the confusion matrix) of predicting the proper target, with a different number of possible targets (Figure 2A). Again, ERGO was not trained for this task, but for the TPP-I task. The targets were the peptides with the highest number of binding TCR in the databases (Supplementary Table S6). The AE produces better accuracies than the LSTM and the prediction for the AE and VDJdb yields better accuracies than McPAS. An important result is that the accuracy is still at 0.5 even for 10 peptides, suggesting that high accuracy can be obtained even when choosing from a large number of peptides.

### Distinguishing TCRs Binding Different Targets (TPP Task)

A more important task from a diagnostic point of view would be to distinguish between TCRs binding different peptides for any set of either known or previously unseen TCRs and peptides. To test the specificity of the prediction, we evaluated ERGO's AUC on the three TPP tasks. The easiest task (TPP-I) is predicting unknown TCR-peptide parings (AE AUC value 0.86). A more complex task is the prediction of pairs containing a known peptide with an unknown TCR (TPP-II—AE AUC value 0.81). The hardest pairing task is to predict the binding of a previously unseen peptide and a previously TCR (TPP-III). This task has never been tested and reaches an AUC of 0.669 (Table 4 and Figure 2B). We now plan to enlarge ERGO to include the alpha chain and V and MHC to see if this score can be improved.

**Table 4:** AUC of TPP task with either known peptide and TCR (but unknown pairing TPP-I), known peptide unseen TCR (TPP-II), and unseen peptide and TCR (TPP-III).

**Evaluation AUC**	**McPAS**		**VDJdb**		**McPAS+ VDJdb**		**Tumor**	
	**AE**	**LSTM**	**AE**	**LSTM**	**AE**	**LSTM**	**AE**	**LSTM**
TPP-I	**0.860**	0.859	0.840	0.842	0.776	0.761	0.805	0.813
TPP-II	**0.810**	0.798	0.792	0.764	0.770	0.745	0.805	0.813
TPP-III	0.601	0.562	0.669	0.522	0.636	**0.674**	0.570	0.646

*The results are the test AUC using either AE or LSTM on McPAS (23) and VDJdb (27) separately or on the joined dataset. The final column is the prediction of tumor antigens TCR binding. Again, the AE consistently outperforms the LSTM, except for the TPP-III task, where increasing the training set size and the complexity of the encoders improves the performance. Bolded values are the best results*.

To test if this performance can be improved by enlarging the training set to better learn the generic properties, we trained ERGO on McPAS (23) and VDJdb (27) simultaneously. On the TPP-III task, the more complex LSTM encoder reached a higher AUC of 0.674, yet on the TPP-I and TPP-II tasks, the results are better when ERGO is trained separately on McPAS and VDJdb (Table 4). McPAS and VDJdb databases contain different TCR and peptides with different distributions (Supplementary Figure S1), therefore ERGO performance on the combined dataset is often lower, suggesting that further increasing the training set with a similarly distributed set would improve the accuracy.

To further test the effect of the training set size, we subsampled the training set and tested the TPP-I AUC score for different sample sizes. The AUC increased with sample size and did not seem to saturate at the current sample size (Figure 2C). Some peptides have many reported TCRs binding them, while some have a single reported binding TCR (Figure 2E). We tested whether a larger number of reported binding TCR improves accuracy (Figure 2F). Again, a higher number of bound TCRs induces higher prediction AUC, suggesting that larger datasets would further improve ERGO's performance.

### Prediction of TCR-Neoantigen Binding

In the future, ERGO may contribute to the development of TCR-based diagnostic tools. However, it can already be used for the detection of TCRs that bind specific tumor antigens. Given a neoantigen extracted from full genome sequencing of tumors (29, 30) and a target TCR, one could estimate the binding probability of the TCR to such a neoantigen. To test for that, we applied ERGO to neoantigen binding prediction; we used a positive dataset of cancer neoantigen peptides and their matching TCRs, published by Zhang et al. (31), and expanded it with TCR-matching neoantigens in the McPAS-TCR and VDJdb databases. We tested again TPP-I, TPP-II, and TPP-III (Table 4), and got a high AUC for TPP-I and II (above 0.8), and 0.65 for the most complex TPP-III task. A caveat of this analysis may be that it was performed on a comparison of a dataset of TCRs binding neo-antigens and T cells from repertoires of healthy donors. Thus, this is not a direct measurement of the possibility of detecting neo-antigen specific TCRs within a donor.

### Comparison of TPP With Literature

While TPP-III was never previously tested, TPP-II was recently tested by Jurtz et al. (21), who used a convolutional neural network (CNN) based model, NetTCR, for predicting binding-probabilities of TCR-HLA-A^*^02:01 restricted peptide pairs. An IEDB dataset was used to train the model. The MIRA assay provided by Klinger et al. (32) was used for evaluating the model by testing the model performance on shared IEDB and MIRA peptides and new TCRs. Jurtz et al. used two models in their experiments. One was trained with positive IEDB examples and only negative examples made from the IEDB dataset itself (no additional sources) while another model had also additional naïve negatives (33). We used the united IEDB and MIRA dataset provided by Jurtz et al. and created also negative examples from that dataset. We trained ERGO models with 80% of the united data (positive and negative examples) and evaluated the model performance on the rest of the data (20%). Again, ERGO outperformed the current results, 0.88 vs. 0.73 (Supplementary Table S3). Note that some differences exist between the training and test set used here and in the Jurtz paper, as detailed in the methods section.

### CDR3 Sequence Characteristics

To test which position along the CDR3 has the strongest effect on the binding prediction, we trained ERGO ignoring one TCR amino-acid position at a time, by nullifying the position in the autoencoder based model or by skipping that position input in the LSTM based model (Figure 2D). Omitting each one of the central amino-acids of the TCR's CDR3 beta (positions 7–15) impairs the model's performance, especially in the LSTM-based model. The autoencoder-based model is more stable than the LSTM based model, perhaps due to exposure to a variety of TCRs in the TCR autoencoder pre-training.

## Discussion

We propose a set of standard tests to evaluate the accuracy of TCR-peptide binding and show that training a model using a combination of deep learning methods and curated datasets on the complex task of pairing random peptides and TCR can lead to high accuracy on all other tests. The main element affecting prediction accuracy is the training size. Enlarging the database improves the prediction accuracy for unseen peptides. Also, when subsampling the existing datasets, the accuracy increases with sample size and does not seem to saturate at the current sample size (Figure 2C).

Several other elements can affect the results, such as the V and J gene used and the alpha chain. In general, TCR-sequencing has often been limited to the TCR β chain due to its greater combinatorial and junctional diversity (10) and to the fact that a single TCRβ chain can be paired with multiple TCRα chains (34). Pogorelyy et al. (35) have shown concordance between TCRα and TCRβ chain frequencies specific for a given epitope and suggested this justifies the exclusive use of TCRβ sequences in analyzing the antigen-specific landscape of heterodimeric TCRs. Only recently, with single-cell techniques that enable pairing of α and β chains sequences, more data on alpha-beta TCRs is accumulating (36). Once large-scale curated alpha-beta TCR-peptide datasets are available, their integration into the current method is straight forward.

ERGO is based on LSTM networks to encode sequential data. Previous models by Jurtz et al. (21) used convolutional neural networks (CNN) for a similar task. While CNNs are good at extracting position-invariant features, RNN (in particular LSTM) can catch a global representation of a sequence, in various NLP tasks (37). Similarly, we did not use attention-based models (38) since the TCR can bind the peptide MHC at different angles and specific TCR positions are not well-correlated with specific peptide positions (39).

ERGO randomly initializes our amino-acid embeddings and trains the embeddings with the model parameters. Using word-embedding algorithms such as Word2Vec (40) or GloVe (41) can give a good starting point to the embeddings. Special options for amino-acids pre-trained embeddings include the use of BLOSUM matrix (42) or Kidera-factors-based manipulations (43). As pre-trained embedding usually provides better model results, we plan to further test such encodings.

The prediction method presented here can serve as a first step in identifying neoantigen-reactive T cells for adoptive cell transfer (ACT) of tumor-infiltrating lymphocytes (TILs) targeting neoantigens (44). The ERGO algorithm can accelerate the preliminary selection of valid target epitopes and corresponding TCRs for adoptive cell transfer. Finally, an important future implication would be to predict TCR-MHC binding, such prediction can be crucial for improving mismatched bone marrow transplants (45).

## Materials and Methods

### Data

Three TCR-peptide datasets were used in the binding prediction task. McPAS-TCR dataset was downloaded from http://friedmanlab.weizmann.ac.il/McPAS-TCR/ and VDJdb dataset was downloaded from https://vdjdb.cdr3.net/, both in November 2019. We used a dataset of cancer neoantigen peptides and their matching TCRs, published by Zhang et al. (31). A set of cancerous peptides was made for extracting TCRs matching to these peptides also in McPAS-TCR and VDJdb databases. We extended the original cancer dataset to include all TCRs-cancerous peptide pairs in all datasets. The data were processed into TCR-peptide pair files, using only TCRβ chains and valid TCR and peptide sequences.

The TCR autoencoder was trained on a data which was derived from a prospective clinical study (NCT00809276) by Kanakry et al. (46) The dataset is freely available at the Adaptive database (www.adaptivebiotech.com) that provides open access to a variety of datasets of TCRs next-generation sequencing.

### Datasets Studied

In each model, training data was loaded as batches of positive and negative examples. For the positive examples, we took the existing TCR-peptide pairs in the database and split it into a train set and a test set. For creating the negative examples for the TPP-I task, we first chose a peptide randomly from the peptides in the training set. Then, we chose five random TCRs from the training set that are not reported to bind this peptide, to create five internal wrong pairs. A similar process was done to create a test set containing positive and negative examples. Thus, the number of negative examples is five times larger than the number of positive examples in both train and test sets.

### Models

We used two models for predicting TCR-peptide binding. The models use deep-learning architectures to encode the TCR and the peptide. Then the encodings are fed into a multilayer perceptron (MLP) to predict the binding probability. Two encoding methods are applied—LSTM acceptor encoding and Autoencoder-based encoding. The peptide is always encoded using the LSTM acceptor method, so the two models differ in the TCR encoding method.

#### LSTM Acceptor

First, the amino acids were embedded using an embedding matrix. We set each amino acid an embedding vector, randomly initialized. Next, the TCR or the peptide was fed into a Long Short Term Memory (LSTM) network as a sequence of vectors. The LSTM network outputs a vector for every prefix of the sequence; we used the last output as the encoding of the whole sequence. We used two different embedding matrices and LSTM parameters for the TCRs and the peptides encodings. The embedding dimension of the amino acids was 10. We use two-layered stacked LSTM, with 500 units at each layer. A dropout rate of 0.1 was set between the layers.

#### TCR Autoencoder

The TCR autoencoder was trained before training the Autoencoder-based attachment prediction model. To train the TCR autoencoder, first we added a “stop-codon” at the end of every TCR CDR3 sequence. Each amino acid was represented as a one-hot vector of 21 numbers (20 possible amino acids and an additional stop codon) where all values were set to zeros except one index of the corresponding amino acid which was set to 1. Each of the CDR3 vector representations one-hot vectors (i.e., 20 positions for each amino acid with zeros, except for the position appropriate for this amino acid) were joined and, terminated with a “stop codon” one-hot vector. Zero padding was then added to the CDR3 vectors, completing the vectors to the maximum lengths chosen according to the data lengths distribution. Each zero codon was represented as a fully zeroed one-hot vector.

The concatenated TCR vectors were fed into the Autoencoder network, which was based on a combination of linear layers, creating similar “encoder” and “decoder” networks. In the encoder, the TCRs were first put into a layer of 300 units, then into a layer of 100 units, and then into the encoding layer of 100 units. This layer output was used to encode the TCR in the trained autoencoder model. We used Exponential Linear Unit (ELU) activation between the linear layers and dropout rate of 0.1. The decoding layers were similar to the encoding layers in the reverse order—first, the encoded TCR vectors were fed into a layer with 100 units, then into a layer with 300 units, and then into a layer with the original TCR concatenated one-hot vector length units. We used softmax (a function translating the last layer into a probability function) on the last decoder layer output on every sub-vector matching to an input amino acid one-hot vector position.

We used Mean Squared Error (MSE) loss (when the decoder output should be like the concatenated one-hot input). The autoencoder was trained using Adam optimizer with a learning rate of 1e-4, we used batching with batch size 50. The autoencoder was trained for 300 epochs.

In order to read the TCR from the decoding vector, we first split the long vector into “one-hot” like vectors. We back-translated the one-hot vectors into amino-acids by taking the amino acid matching to the maximal value index in the vector (which should be 1). We dropped all amino acids from the stop codon and forward to get a sequence of amino acid which should be the TCR. The autoencoder was trained with 80% of the data and was evaluated with the rest of it. The autoencoder was only trained on the TCRs and no information on the peptides was ever used to train the autoencoder.

#### MLP Classifier (Also Mentioned as FFN)

In both models, the TCR encoding was concatenated to the peptide encoding and fed into the MLP. The MLP contains one hidden layer with as units as half of the concatenated vector size and sigmoid is used on the output of the last layer to get a probability value. In both models, the activation in the MLP is Leaky ReLU. Dropout with a rate of 0.1 was set between layers.

#### Model Configurations

As mentioned, we used two models, the LSTM based model and the autoencoder based model. We trained the embeddings, the LSTM parameters and the MLP in the first model, and the TCR autoencoder, peptide LSTM encoder and MLP parameters in the second model. The trained TCR autoencoder parameters were loaded to the autoencoder based model and are trained again within all model parameters.

We used Binary Cross Entropy (BCE) loss. Since we get five times more negative samples than positive samples according to the described sampling method, the loss is weighted, respectively, by a factor of 5/6 for positive samples and by 1/6 for negative samples. The optimizer was Adam with a learning rate of 1e-3 and weight decay 1e-5. We used batching with batch size 50. The model was trained for 100 epochs. The models used 80% of the data for training and 20% for evaluation for all datasets.

All models were implemented with PyTorch library in Python programming language.

The prediction models were evaluated using Area Under the Curve (AUC) score.

#### Hyperparameters Tuning

Both LSTM based model and the Autoencoder based model hyperparameters were optimized using a grid search in the hyperparameters space. The hyperparameters to optimize were the embedding matrix dimension, the LSTM dimensions, learning rate, weight decay, activation functions, etc. All models were tested with the same grid search. Once the grid search was finished, we chose five new sets of training and test set and reported their results. Note that the results are quite robust to most parameter changes, and that the size of the TCR-peptide pair space is much larger than any of the training sets used during parameter tuning.

### Experiments Configuration

At the broad level, the ERGO model was trained and designed to solve the TPP-I task. Since the train and the test set are chosen randomly for each training process (as described above), 5 trained models along with their matching train and test set were analyzed, for each database [McPAS (23) or VDJdb (27)] and model type (LSTM based or AE based). Train and test sizes are detailed in Supplementary Table S2.

#### Single Peptide Binding

For computing single-peptide binding score, samples from each test set were observed. For every peptide, we looked for the pairs in the test set containing that peptide (positive and negative samples). The ROC and AUC scores were computed according to the model prediction of those pairs. SPB scores were computed for the five most frequent peptides in each database (Table 1) and three human peptides appearing in Dash et al. (18) dataset (Table 2). Results for peptides with more than 50 reported binding TCRs are in the Supplementary Table S4. Mean AUC scores are reported. Test sizes for the SPB task are detailed in Supplementary Table S7.

Single protein scores are extracted similarly, by analyzing all pairs in the test set that contain a peptide of the specific protein.

#### Multi-Peptide Selection

At first, the number of classes k was set. Trained model prediction scores were extracted for each TCR in the test set, paired with every peptide from the top k frequent peptides in the relevant database. The TCR target was predicted to be the peptide which got the maximal score as the pair complement. Accuracy was computed using the true samples in the test set. This was done for class numbers ranging between 2 and 10, as well as 20 and 30. Mean accuracies are shown in Figure 2A. Test sizes for the MPS task are detailed in Supplementary Table S8.

#### TCR-Peptide Pairing

New test TCRs and new test peptides were deducted from the train and test sets. TPP-I score is the AUC of the model predictions of the original test set. TPP-II is the AUC of the predictions of the new test TCR positive and negative samples. TPP-III is the AUC of the predictions of new test TCR and new test peptide pairs, positive and negative samples.

#### Train Data Sub-sampling

All models were trained and evaluated using the same train and test partition. Every model train set was a sub-sample of the original train set, while the test set remained the same. Ten thousand new train samples were added at each iteration.

#### Missing Positions Training

Again, all models were evaluated with the same test set and a train/test partition. In this experiment, the train data was modified by dropping a single amino acid in a specific position at a time. Practically, this was done by deleting this position for all TCRs in the LSTM based model, or by nullifying the relevant position in the one-hot encoding of the TCRs in the AE based model. This experiment was repeated five times, Mean TPP-I scores are shown in Figure 2D.

#### TPP Per-Number of TCRs Per-Peptide

First, TCR records per peptide were counted in the original McPAS (23) and VDJdb (27) databases. Given a test set, the test pairs were divided into bins, according to the number of TCR records per peptide in the original database. The differences between the bins were on an exponential scale. AUC score was computed for each bin. Mean AUC scores are shown in Figure 2F.

#### Comparison With NetTCR

The united IEDB and MIRA datasets were downloaded from https://github.com/mnielLab/netTCR. Unfortunately, the authors did not publish the IEDB train data separated from the MIRA test data, thus we had to evaluate ERGO in another train/test partition. We used 80% of the IEDB and MIRA data for training and the rest of it (20%) for testing. Additional “C” prefix and “F” suffix were added to each TCR sequence. The MIRA data was containing new test TCRs (but was not evaluated with new test peptides), therefore we compare NetTCR results with ERGO TPP-II scores (Supplementary Table S3).

## Data Availability Statement

Publicly available datasets were analyzed in this study. This data can be found here: http://friedmanlab.weizmann.ac.il/McPAS-TCR/ and https://vdjdb.cdr3.net/.

## Author Contributions

IS developed the formalism and implemented it. SD designed the TCR autoencoder formalism. NT-M developed the libraries and wrote the manuscript. YL supervised the work and wrote the manuscript. HB designed the initial formalism. All authors contributed to the article and approved the submitted version.

## Conflict of Interest

The authors declare that the research was conducted in the absence of any commercial or financial relationships that could be construed as a potential conflict of interest.
