# Supplementary material for: Prediction of Specific TCR-Peptide Binding From Large Dictionaries of TCR-Peptide Pairs
Source: Front Immunol. 2020 Aug 25;11:1803. doi: 10.3389/fimmu.2020.01803 (PMC7477042; doi:10.3389/fimmu.2020.01803)

Supplementary Material

1 Supplementary Figures and Tables

Table S1: *TCR autoencoder accuracy per number of mismatches allowed in sequence decoding.*

Number of mismatches allowed	Autoencoder Accuracy
0	0.920
1	0.982
2	0.993

Table S2: *TPP Train and test dataset sizes of all trained models for the different versions of the ERGO classifier (AE vs. LSTM and McPAS[26] vs VDJdb[27]).*

Iteration	Database	McPAS				VDJdb			
	Model type	AE		LSTM		AE		LSTM	
	Dataset	Train	Test	Train	Test	Train	Test	Train	Test
1	TCR/Peptide pairs	66985	17316	67646	16659	209713	52758	209779	52693
	Positive pairs	11164	2886	11274	2776	34952	8793	34963	8782
	Negative pairs	55821	14430	56372	13883	174761	43965	174816	43911
	New test TCRs		2119		2047		5852		5873
	TPP-II test size		13607		13043		37714		37554
	New test peptides		14		28		10		5
	TPP-III test size		101		190		56		28
2	TCR/Peptide pairs	67351	16952	67094	17210	211209	51264	209396	53078
	Positive pairs	11225	2825	11182	2868	35201	8544	34899	8846
	Negative pairs	56126	14127	55912	14342	176008	42720	174497	44232
	New test TCRs		2092		2118		5734		5918
	TPP-II test size		13248		13843		36746		37912
	New test peptides		21		15		14		12
	TPP-III test size		91		79		98		66
3	TCR/Peptide pairs	67286	17018	67592	16711	209538	52933	209832	52638
	Positive pairs	11214	2836	11265	2785	34923	8822	34972	8773
	Negative pairs	56072	14182	56327	13926	174615	44111	174860	43865
	New test TCRs		2105		2011		5858		5820
	TPP-II test size		13357		12955		37575		37198
	New test peptides		24		17		15		16
	TPP-III test size		160		120		112		117
4	TCR/Peptide pairs	67644	16658	67489	16814	210232	52245	210440	52035
	Positive pairs	11274	2776	11248	2802	35038	8707	35073	8672
	Negative pairs	56370	13882	56241	14012	175194	43538	175367	43363
	New test TCRs		2076		2064		5804		5803

	**TPP-II test size**		13214		13187		37354		37359
	**New test peptides**		25		24		9		18
	**TPP-III test size**		173		124		62		113
5	**TCR/Peptide pairs**	67332	16968	67014	17290	209670	52802	209365	53108
	**Positive pairs**	11222	2828	11169	2881	34945	8800	34894	8851
	**Negative pairs**	56110	14140	55845	14409	174725	44002	174471	44257
	**New test TCRs**		2088		2122		5895		5914
	**TPP-II test size**		13258		13389		37766		37996
	**New test peptides**		19		20		9		13
	**TPP-III test size**		115		146		62		57

Table S3: *Comparison between the different versions of the ERGO classifier (AE vs. LSTM) and existing NetTCR[21] models. TPP-II results are shown.*

**Model**	**Train data**	**Test data**	**TPP-II score**
ERGO AE	IEDB + MIRA (80%)	IEDB + MIRA (20%)	0.886
ERGO LSTM	IEDB + MIRA (80%)	IEDB + MIRA (20%)	0.883
NetTCR INT_NEG	IEDB	MIRA (shared IEDB peptides)	0.697
NetTCR ADD_NEG	IEDB + additional negatives	MIRA (shared IEDB peptides)	0.727

Table S4: *Comparison between the different versions of the ERGO classifier (AE vs. LSTM and McPAS[26] vs VDJdb[27]) for the SPB task. Peptides with more than 50 TCRs in each database are shown. The values are the AUC over the test set of unseen TCR for this peptide.*

**McPAS**			**VDJdb**		
**Model**	**AE**	**LSTM**	**Model**	**AE**	**LSTM**
**Peptide**			**Peptide**		
LPRRSGAAGA	0.772431	0.767037	KLGGALQAK	0.695061	0.731208
GILGFVFTL	0.843091	0.832766	GILGFVFTL	0.820975	0.817835
NLVPMVATV	0.835317	0.821623	NLVPMVATV	0.665098	0.686264
GLCTLVAML	0.803314	0.816092	AVFDRKSDAK	0.676548	0.695028
SSYRRPVGI	0.969659	0.980026	RAKFKQLL	0.828297	0.825514
RFYKTLRAEQASQ	0.967792	0.936695	ELAGIGILTV	0.735488	0.862051
SSLENFRAYV	0.942426	0.942475	GLCTLVAML	0.764231	0.770183
WEDLFCDESLSSPEPPSSSE	0.901104	0.920351	IVTDFSVIK	0.763487	0.764299
CRVLCCYVL	0.770348	0.768169	SSYRRPVGI	0.989022	0.986433
ASNENMETM	0.940957	0.944221	SSLENFRAYV	0.969169	0.970508
ELAGIGILTV	0.828654	0.816382	RLRAEAQVK	0.726325	0.695372
LLWNGPMAV	0.856099	0.838728	TTPESANL	0.966307	0.970118
VTEHDTLLY	0.810553	0.815736	CTPYDINQM	0.959594	0.968356
TPRVTGGGAM	0.803075	0.787879	LLWNGPMAV	0.725481	0.75429
HGIRNASFI	0.959277	0.959055	HGIRNASFI	0.971845	0.981121

EAAGIGILTV	0.854633	0.85533	ASNENMETM	0.974924	0.976743
FRCPRRFCF	0.824175	0.790418	PKYVKQNTLKLAT	0.691382	0.663138
VEALYLVCG	0.907081	0.935765	FRDYVDRFYKTLRAEQASQE	0.952822	0.936532
LSLRNPILV	0.902143	0.924408	KRWIILGLNK	0.842182	0.837442
RPHERNGFTVL	0.714755	0.731987	SSPPMFRV	0.984529	0.985437
SSPPMFRV	0.976377	0.977021	STPESANL	0.969857	0.974328
RAKFKQLL	0.832942	0.814849	LSLRNPILV	0.93783	0.963003
KAFSPEVIPMF	0.843734	0.913372	CINGVCWTV	0.813524	0.827376
NLNCCSVPV	0.850514	0.789345	TPRVTGGGAM	0.845308	0.841128
MEVGWYRSPFSRVVHLYRNGK	0.987286	0.983272	LLLGIGILV	0.728453	0.682906
KRWIILGLNK	0.767641	0.766451	KAFSPEVIPMF	0.840425	0.902281
FPRPWLHGL	0.795516	0.884843	ATDALMTGY	0.884728	0.83388
TVYGFCLL	0.874119	0.911252	VTEHDTLLY	0.625833	0.639702
KMVAVFYTT	0.757869	0.79095	EIYKRWII	0.775054	0.768612
RPRGEVRFL	0.862574	0.922006	FLKEKGGL	0.738713	0.747265
ATDALMTGY	0.941682	0.869135	GTSGSPIVNR	0.84617	0.854691
YVLDHLIVV	0.878755	0.7948	TVYGFCLL	0.970757	0.972494
VVLSWAPPV	0.710898	0.721487	GTSGSPIINR	0.862618	0.864304
YSEHPTFTSQY	0.868444	0.719544	SQLLNAKYL	0.987209	0.985011
HPKVSSEVHI	0.840526	0.846167	LPRRSGAAGA	0.619644	0.585148
			NLSALGIFST	0.707337	0.756388
			RPRGEVRFL	0.89807	0.951702
			ARMILMTHF	0.920818	0.9468
			SFHSLHLLF	0.736924	0.7445
			QARQMVQAMRTIGTHP	0.650485	0.665243
			KAVYNFATC	0.894006	0.932316
			GLIYNRMGAVTTEV	0.642705	0.606565
			TPQDLNTML	0.752846	0.737685
			IPSINVHHY	0.75858	0.74078
			DPFRLLQNSQVFS	0.67448	0.747417
			YVLDHLIVV	0.678381	0.723087
			YSEHPTFTSQY	0.802649	0.869689
			KLVALGINAV	0.634441	0.718725
			RTLNAWVKV	0.555511	0.672836
			FLRGRAYGL	0.90147	0.843084
			FPRPWLHGL	0.865248	0.760968
			AMFWSVPTV	0.591343	0.707997
			TPGPGVRYPL	0.797351	0.747012
			KRWIIMGLNK	0.853095	0.828291
			AYAQKIFKI	0.598942	0.595577
			SLFNTVATLY	0.677828	0.682458
			FLYALALLL	0.878	0.883727
			GPGHKARVL	0.544828	0.742347
			LLFGYPVYV	0.614185	0.666065

			MLNIPSINV	0.583162	0.635572
			SLYNTVATL	0.795793	0.77279
			NEGVKAAW	0.817963	0.724294
			FLASKIGRLV	0.669265	0.721543
			QVPLRPMTYK	0.821861	0.849146
			SGPLKAEIAQRLED	0.746869	0.631266
			EPLPQGQLTAY	0.880626	0.85187
			SYIGSINNI	0.965756	0.954853
			HPVGEADYFEY	0.94127	0.89122
			FLYNLLTRV	0.620185	0.708811
			NAITNAKII	0.968216	0.938254
			GTSGSPIIDK	0.72907	0.733715
			ISPRTLNAW	0.686906	0.722699
			HPKVSSEVHI	0.847606	0.73928
			RPPIFIRRL	0.803643	0.822873
			LLDFVRFMGV	0.773148	0.634493
			QYDPVAALF	0.617483	0.624344

Supp. Mat. Table S5: *Comparison between the different versions of the ERGO classifier (AE vs. LSTM and McPAS[26] vs VDJdb[27]) and existing classifiers (TCRGP by Jokinen et al.[19], TCRex by Gielis et al.[20]) for the SPB task. The peptides are those from VDJdb tested by Jokinen et al. The values are the AUC over the test set of unseen TCR for this peptide.*

**Peptide**	**ERGO**		**TCRGP (β,3)**		**TCRex**
	**AE**	**LSTM**	**LOSO**	**unique LOO**	
IPSINVHHY	0.758	0.74	0.852	0.797	0.84±0.04
TPRVTGGGAM	0.845	0.841	0.892	0.768	0.88±0.03
NLVPMVATV	0.665	0.686	0.912	0.838	0.72±0.01
GLCTLVAML	0.764	0.77	0.926	0.782	0.82±0.08
RAKFKQLL	0.828	0.825	0.887	0.729	0.89±0.01
YVLDHLIVV	0.678	0.723	0.682	0.541	0.76±0.07
GILGFVFTL	0.82	0.817	0.881	0.804	0.81±0.01
PKYVKQNTLKLAT	0.691	0.663	0.706	0.563	0.72±0.02
CINGVCWTV	0.813	0.827	0.819	0.887	0.75±0.06
KLVALGINAV	0.634	0.718	0.695	0.484	0.73±0.08
ATDALMTGY	0.884	0.833	0.678	0.677	0.91±0.04
RPRGEVRFL	0.897	0.951	0.801	0.694	0.92±0.05
LLWNGPMAV	0.725	0.754	0.825	0.813	0.79±0.01
GTSGSPIVNR	0.846	0.854	0.864	0.832	0.88±0.04
GTSGSPIINR	0.855	0.864	0.734	0.736	0.86±0.03
KAFSPEVIPMF	0.84	0.902	0.769	0.755	0.9±0.01
TPQDLNTML	0.752	0.737	0.798	0.742	0.94±0.04
EIYKRWII	0.775	0.768	0.75	0.904	0.75±0.06
KRWIILGLNK	0.839	0.837	0.702	0.535	0.85±0.04

FRDYVDRFYKTLRAEQASQE	0.952	0.936	0.893	0.822	1.0±0.0
GPGHKARVL	0.544	0.742	0.838	0.803	-
FLKEKGGL	0.738	0.747	0.817	0.766	0.76±0.06

Supp. Mat. Table S6*: The peptides with the highest number of binding TCRs in McPAS[26] and VDJdb[27] databases, in decreasing order. The 30 most frequent peptides in both databases are shown.*

**McPAS**		**VDJdb**	
**Peptide**	**TCRs**	**Peptide**	**TCRs**
LPRRSGAAGA	2145	KLGGALQAK	27842
GILGFVFTL	1920	GILGFVFTL	7008
NLVPMVATV	1134	NLVPMVATV	4991
GLCTLVAML	1091	AVFDRKSDAK	3534
SSYRRPVGI	653	RAKFKQLL	2720
RFYKTLRAEQASQ	602	ELAGIGILTV	2519
SSLENFRAYV	549	GLCTLVAML	1599
WEDLFCDESLSSPEPPSSSE	477	IVTDFSVIK	1480
CRVLCCYVL	435	SSYRRPVGI	1306
ASNENMETM	427	SSLENFRAYV	866
ELAGIGILTV	325	RLRAEAQVK	860
LLWNGPMAV	307	TTPESANL	859
VTEHDTLLY	280	CTPYDINQM	814
TPRVTGGGAM	279	LLWNGPMAV	697
HGIRNASFI	279	HGIRNASFI	558
EAAGIGILTV	278	ASNENMETM	536
FRCPRRFCF	266	PKYVKQNTLKLAT	484
VEALYLVCG	207	FRDYVDRFYKTLRAEQASQE	471
LSLRNPILV	203	KRWIILGLNK	413
RPHERNGFTVL	191	SSPPMFRV	326
SSPPMFRV	163	STPESANL	309
RAKFKQLL	144	LSLRNPILV	290
KAFSPEVIPMF	143	CINGVCWTV	284
NLNCCSVPV	128	TPRVTGGGAM	273
MEVGWYRSPFSRVVHLYRNGK	128	LLLGIGILV	233
KRWIILGLNK	99	KAFSPEVIPMF	227
FPRPWLHGL	88	ATDALMTGY	207
TVYGFCLL	87	VTEHDTLLY	203
KMVAVFYTT	74	EIYKRWII	176
RPRGEVRFL	65	FLKEKGGL	175

***Supp. Mat. Table S7**: Single peptide binding (SPB) test sizes of all trained models for the different versions of the ERGO classifier (AE vs. LSTM and McPAS[26] vs VDJdb[27]). This table matches the results reported in Table 1 and Table 2.*

**Iteration**	**Database**	**Peptide**	**McPAS**		**Peptide**	**VDJdb**		**Peptide**	**McPAS + VDJdb**	
	**Model type**		**AE**	**LSTM**		**AE**	**LSTM**		**AE**	**LSTM**
**1**	**Positive test samples**	LPRRSGAAGA	435	403	KLGGALQAK	2791	2775	GLCTLVAML	472	476
	**Negative test samples**		1390	1290		4585	4566		2682	2654
	**Positive test samples**	GILGFVFTL	345	315	GILGFVFTL	1001	983	NLVPMVATV	1129	1124
	**Negative test samples**		1222	1122		4474	4253		5012	5133
	**Positive test samples**	NLVPMVATV	187	164	NLVPMVATV	941	992	GILGFVFTL	1315	1272
	**Negative test samples**		929	750		4237	4372		5703	5629
	**Positive test samples**	GLCTLVAML	200	220	AVFDRKSDAK	355	338			
	**Negative test samples**		914	985		2230	2248			
	**Positive test samples**	SSYRRPVGI	130	146	RAKFKQLL	284	284			
	**Negative test samples**		641	665		1932	2010			
**2**	**Positive test samples**	LPRRSGAAGA	404	428	KLGGALQAK	2719	2881	GLCTLVAML	475	460
	**Negative test samples**		1281	1353		4495	4537		2793	2805
	**Positive test samples**	GILGFVFTL	336	312	GILGFVFTL	985	978	NLVPMVATV	1140	1086
	**Negative test samples**		1225	1201		4290	4430		4997	5007
	**Positive test samples**	NLVPMVATV	192	169	NLVPMVATV	924	947	GILGFVFTL	1351	1292
	**Negative test samples**		929	827		4060	4325		5654	5515
	**Positive test samples**	GLCTLVAML	211	217	AVFDRKSDAK	337	343			
	**Negative test samples**		918	960		2080	2184			
	**Positive test samples**	SSYRRPVGI	137	148	RAKFKQLL	270	307			
	**Negative test samples**		687	753		1948	2058			
**3**	**Positive test samples**	LPRRSGAAGA	436	420	KLGGALQAK	2792	2793	GLCTLVAML	438	456
	**Negative test samples**		1352	1248		4603	4587		2632	2654
	**Positive test samples**	GILGFVFTL	321	356	GILGFVFTL	1025	975	NLVPMVATV	1159	1107
	**Negative test samples**		1212	1302		4475	4348		5274	4950
	**Positive test samples**	NLVPMVATV	177	179	NLVPMVATV	975	1008	GILGFVFTL	1337	1292
	**Negative test samples**		854	878		4417	4421		5633	5674
	**Positive test samples**	GLCTLVAML	232	234	AVFDRKSDAK	359	337			
	**Negative test samples**		1022	933		2173	2100			
	**Positive test samples**	SSYRRPVGI	136	139	RAKFKQLL	301	290			
	**Negative test samples**		733	752		2114	1823			
**4**	**Positive test samples**	LPRRSGAAGA	437	418	KLGGALQAK	2764	2764	GLCTLVAML	507	460
	**Negative test samples**		1354	1313		4500	4511		2945	2705
	**Positive test samples**	GILGFVFTL	330	325	GILGFVFTL	959	980	NLVPMVATV	1184	1125
	**Negative test samples**		1214	1217		4232	4360		5254	5059
	**Positive test samples**	NLVPMVATV	169	172	NLVPMVATV	959	960	GILGFVFTL	1288	1340
	**Negative test samples**		735	879		4322	4272		5675	5673
	**Positive test samples**	GLCTLVAML	204	186	AVFDRKSDAK	346	329			
	**Negative test samples**		936	848		2172	2103			
	**Positive test samples**	SSYRRPVGI	142	145	RAKFKQLL	317	280			
	**Negative test samples**		748	706		2133	1964			
**5**	**Positive test samples**	LPRRSGAAGA	415	456	KLGGALQAK	2800	2848	GLCTLVAML	486	465
	**Negative test samples**		1325	1432		4589	4571		2774	2624
	**Positive test samples**	GILGFVFTL	319	314	GILGFVFTL	996	986	NLVPMVATV	1126	1131
	**Negative test samples**		1199	1214		4435	4426		5174	5066
	**Positive test samples**	NLVPMVATV	172	173	NLVPMVATV	985	949	GILGFVFTL	1250	1307
	**Negative test samples**		815	808		4416	4248		5448	5639
	**Positive test samples**	GLCTLVAML	219	231	AVFDRKSDAK	372	342			
	**Negative test samples**		984	949		2485	2132			
	**Positive test samples**	SSYRRPVGI	132	134	RAKFKQLL	308	291			
	**Negative test samples**		655	688		2207	1999			

***Supp. Mat. Table S8**: Multi Peptide Selection (MPS) test sizes of all trained models for the different versions of the ERGO classifier (AE vs. LSTM and McPAS[26] vs VDJdb[27]). This table matches the results reported in Figure 2A.*

**Iteration**	**Number of peptides**	**McPAS**		**VDJdb**	
		**AE**	**LSTM**	**AE**	**LSTM**
**1**	**2**	624	558	3096	3164
	**3**	763	708	3732	3751
	**4**	871	809	3913	3948
	**5**	951	898	4082	4094
	**10**	1271	1200	4580	4546
	**20**	1633	1548	5131	5096
	**30**	1796	1707	5393	5366
**2**	**2**	586	610	3030	3169
	**3**	744	767	3645	3755
	**4**	858	861	3817	3921
	**5**	936	942	3977	4076
	**10**	1268	1266	4448	4580

	**20**	1621	1638	4953	5104
	**30**	1770	1805	5242	5405
**3**	**2**	607	605	3143	3136
	**3**	772	771	3783	3722
	**4**	871	880	3961	3895
	**5**	964	1037	4118	4056
	**10**	1265	1268	4604	4546
	**20**	1642	1591	5116	5058
	**30**	1787	1726	5385	5343
**4**	**2**	619	591	3091	3067
	**3**	780	719	3672	3676
	**4**	876	818	3846	3842
	**5**	963	899	4013	4007
	**10**	1263	1212	4512	4505
	**20**	1614	1566	5010	5023
	**30**	1759	1717	5308	5301
**5**	**2**	585	618	3174	3181
	**3**	745	774	3792	3795
	**4**	837	882	3987	3963
	**5**	924	970	4518	4126
	**10**	1249	1262	4635	4611
	**20**	1614	1624	5148	5144
	**30**	1763	1786	5419	5427

Supp. Mat. Table S9*: An index to each train and test size for the different suggested tests. The training set is always the training set for the TPP-I task.*

**Task**	**Train size**	**Test description**	**Test size**
**SPB**	Table S2 Train TCR/Peptide pairs	TPP-II (depends on specific peptide)	Table S7
**MPS**		TPP-II (depends on number of peptides)	Table S8
**TPP-I**		TPP-I (new TCR-peptide pairs)	Table S2 Test TCR/Peptide pairs
**TPP-II**		TPP-II (new TCRs paired with known peptides)	Table S2 TPP-II test size
**TPP-III**		TPP-III (new TCRs paired with new peptides)	Table S2 TPP-III test size

Figure S1*: Differences between McPAS[26] and VDJdb[27] datasets. A) Normalized TCR length distribution in McPAS and VDJdb datasets. B) Normalized peptide length distribution in McPAS and VDJdb datasets. C) T cell type distribution (CD4/CD8) of TCRβ in McPAS and VDJdb datasets. D) Number of TCRs per peptide distribution, for unique TCRs in McPAS-TCR, unique TCRs in VDJdb, and common TCRs, logarithmic scale.*

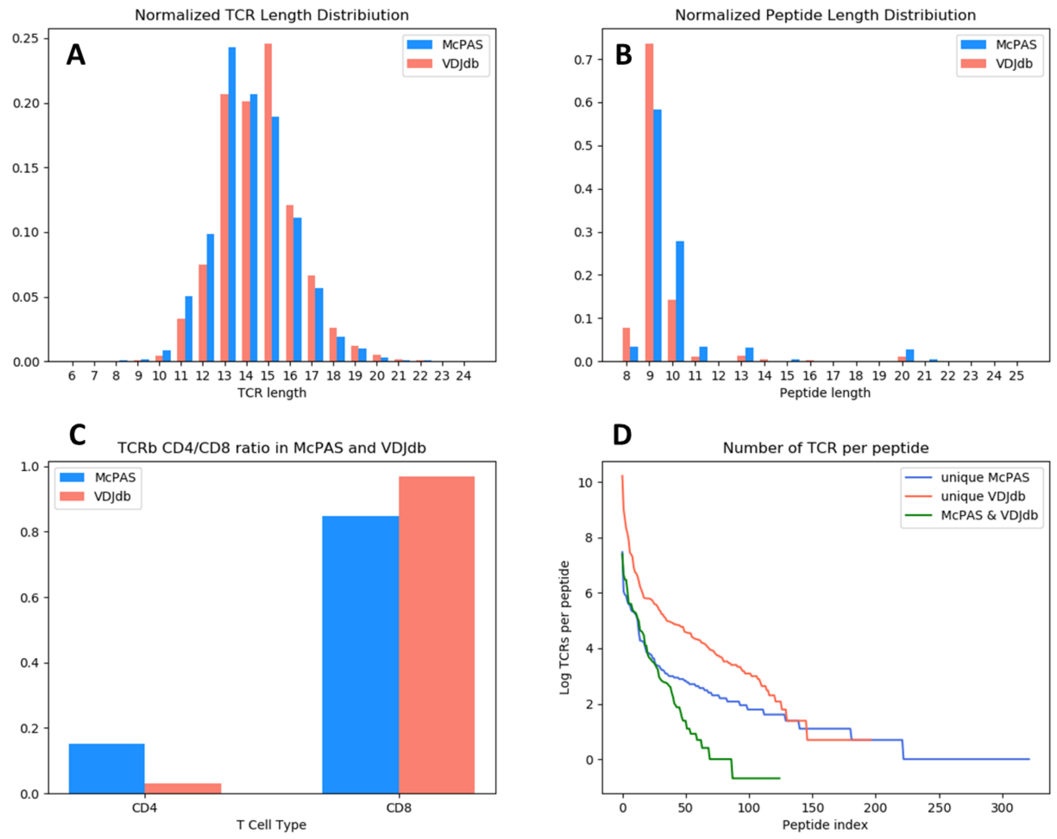

Supplement: Supplementary file 1 [file Presentation_1.pdf]
